# Characterization of somatosensory neuron involvement in the SOD1^G93A^ mouse model

**DOI:** 10.1038/s41598-022-11767-8

**Published:** 2022-05-09

**Authors:** Miguel A. Rubio, Mireia Herrando-Grabulosa, Nuria Gaja-Capdevila, Jorge J. Vilches, Xavier Navarro

**Affiliations:** 1 Neuromuscular Unit, Department of Neurology, Hospital del Mar, Barcelona, Spain; 2 Department of Cell Biology, Physiology and Immunology, Institute of Neurosciences and CIBERNED, Universitat Autònoma de Barcelona, Bellaterra, Spain; 3 Unitat de Fisiologia Medica, Facultat de Medicina, Universitat Autònoma de Barcelona, 08193 Bellaterra, Spain

**Keywords:** Diseases of the nervous system, Somatosensory system, Neurological disorders

## Abstract

SOD1^G93A^ mice show loss of cutaneous small fibers, as in ALS patients. Our objective is to characterize the involvement of different somatosensory neuron populations and its temporal progression in the SOD1^G93A^ mice. We aim to further define peripheral sensory involvement, analyzing at the same time points the neuronal bodies located in the dorsal root ganglia (DRG) and the distal part of their axons in the skin, in order to shed light in the mechanisms of sensory involvement in ALS. We performed immunohistochemical analysis of peptidergic (CGRP), non-peptidergic (IB4) fibers in epidermis, as well as sympathetic sudomotor fibers (VIP) in the footpads of SOD1^G93A^ mice and wild type littermates at 4, 8, 12 and 16 weeks of age. We also immunolabeled and quantified neuronal bodies of IB4, CGRP and parvalbumin (PV) positive sensory neurons in lumbar DRG. We detected a reduction of intraepidermal nerve fiber density in the SOD1^G93A^ mice of both peptidergic and non-peptidergic axons, compared with the WT, being the non-peptidergic the fewest. Sweat gland innervation was similarly affected in the SOD1^G93A^ mouse at 12 weeks. Nonetheless, the number of DRG neurons from different sensory populations remained unchanged during all stages. Cutaneous sensory axons are affected in the SOD1^G93A^ mouse, with non-peptidergic being slightly more vulnerable than peptidergic axons. Loss or lack of growth of the distal portion of sensory axons with preservation of the corresponding neuronal bodies suggest a distal axonopathy.

## Introduction

Amyotrophic lateral sclerosis (ALS) is a neurodegenerative disorder that mainly involves the motor neurons in the spinal cord and the cerebral cortex. Nevertheless, in the last years, cumulative data have shown that there are other neural structures besides motor neurons that are also affected. Aside cognitive impairments in up to 50% of patients, other non-motor manifestations have been reported, including extrapyramidal, autonomic, and even sensory abnormalities^1,2^. Particularly, a small fiber sensory neuropathy has been observed in several studies in ALS patients^3–6^ and in animal models^7,8^. We have previously reported that in the SOD1^G93A^ mouse, the most common animal model for the disease, there is a significant axonal loss of sensory axons in the skin of the footpads, even at the presymptomatic stage of the disease^9^*,* showing a gradient of involvement from the epidermis (most affected) to the deepest dermis (less affected). However, although peripheral sensory involvement has been confirmed in both humans and animal models, characterization of the neuronal populations most involved, the evolution overtime and the degenerative mechanism are still lacking.

Cutaneous nerve endings are the terminal portions of axons that raise from the body of the pseudo-unipolar neurons located in the dorsal root ganglia (DRG). These neurons and their sensory fibers are classified in subpopulations according to anatomy, physiology, and neurochemistry considerations and present different molecular marker expressions, distinctive receptor characteristics and functions that could have different vulnerability to the neurodegenerative process in ALS. Based on the size and the molecular characteristics, DRG neurons can be divided into three groups: large, myelinated neurons (Aβ mechanoreceptors fibers and Aaβ proprioceptors, the latter expressing parvalbumin; PV), small myelinated (Aδ fibers), and unmyelinated (C fibers) that may be peptidergic neurons (expressing substance P and calcitonin gene-related peptide CGRP) and non-peptidergic (labeled with isolectin B4, IB4) neurons^10,11^. Parvalbumin expressing neurons in DRG are mostly (≥ 90%) proprioceptors, while the rest correspond to other mechanoreceptors^12–14^. Peptidergic and non-peptidergic small sensory neurons share some similarities but have some differences. All form free endings in epidermis and dermis. CGRP + sensory neurons are small myelinated (Aδ type) and unmyelinated (C type), use glutamate as main neurotransmitter and CGRP and some substance P (SP) as neuromodulators, and are regulated during development by nerve growth factor (NGF)^15,16^. Somatic CGRP sensory fibers act as polymodal nociceptors, responding to high-threshold mechanical, thermal, and chemical stimuli^17,18^. Unmyelinated CGRP + fibers project predominantly to laminae I and IIo (outer layer) in the ventral horn of the spinal cord, whereas small myelinated CGRP axons innervate laminae I and III/IV. IB4+ axons correspond to unmyelinated C nociceptors^19^. These neurons are regulated by glial cell line derived neurotrophic factor (GDNF), although they are dependent on NGF until early postnatal stage when they switch dependence to GDNF^20^. IB4+ central projections synapse into the IIi (inner layer) Rexed lamina^21^.

Our objective was to better define the peripheral sensory involvement in the SOD1^G93A^ mice from the epidermal terminations to the soma in the DRG, distinguishing between the different populations of sensory neurons affected. To achieve this aim, we have labeled and quantified the presence of peptidergic and non-peptidergic intraepidermal nerve fiber (IENF), as well as the sweat gland nerve fiber (SGNF) density as a comparative autonomic innervation, at different stages of the disease, and compared with their wild type (WT) littermates. In parallel we have studied the proportion of neuronal somas of the different sensory populations in the DRG.

## Materials and methods

### Transgenic SOD1^G93A^ mice

Transgenic female mice with the G93A human SOD1 mutation [B6SJL-Tg(SOD1-G93A)1Gur] were obtained from Jackson Laboratories, and maintained at the Animal Service of the Universitat Autònoma de Barcelona. The offspring was identified by PCR amplification of DNA extracted from the tail. Non-transgenic littermates were used as controls. All animal experiments conducted were approved by the Ethical Committee of the Universitat Autònoma de Barcelona in accordance with the European Communities Council Directive 2010/63/EU. The study was performed in compliance with the Animal Research Reporting in Vivo Experiments guidelines. The study was carried out in compliance with the ARRIVE guidelines (https://arriveguidelines.org). The experimenter performing the analyses (IENF density, SGNF density and DRG neuron counting) was blind to the group and age of the animals.

The SOD1^G93A^ mice on B6SJL background develop normally until about 12 weeks of age, when they start to show functional deficits in locomotion and muscle strength tests, and weakness progresses until death occurs at an average of 20 weeks, with slight differences between sexes^22,23^. From 8 weeks electrophysiological tests identify clear abnormalities in these mice^24^. Therefore, we used SOD1^G93A^ mice of 4 weeks (normal juvenile), 8 weeks (presymptomatic stage), 12 weeks (early symptomatic stage) and 16 weeks (advanced symptomatic stage).

### Footpad sample collection and processing

Animals were euthanized under anesthesia with pentobarbital (50 mg/kg i.p.) and transcardially perfused with 4% paraformaldehyde in PBS. The plantar pads of the hindfoot of SOD1^G93A^ mice and WT mice were carefully extracted at 4, 8, 12 and 16 weeks of age (4 mice per age and group). Footpads were then kept frozen in a cryoprotective solution (PBS with 30% sucrose and sodium azide). 60 µm-thick sections were serially cut with a cryotome (Leica) and collected by free-floating in PBS medium.

For immunohistochemistry, sections were blocked with PBS-Triton 0.3%-normal donkey serum 1.5% and normal goat serum 10% with 1% bovine serum albumin (BSA) and primary incubated in lectin (unconjugated Griffonia Simplicifolia Lectin I) at 4 °C overnight, and subsequently incubated overnight at 4 °C with anti-protein gene product 9.5 (PGP 9.5, 1:800, Ultraclone), anti-CGRP (1:200, PC205L Millipore), or anti-lectin (1:500, anti-Griffonia Simplicifolia Lectin I, unconjugated, L-1104 Vector) as primary antibodies. Additionally, samples from both SOD1^G93A^ and WT at 4 and 12 weeks were incubated with anti-VIP (1:500, 20077 Immunostar) as a selective marker of the sympathetic innervation of the sweat glands. After washes, sections were incubated overnight at 4 °C with Cy3 (1:200, Jackson Immunoresearch), Alexa 488 (1:200, Thermofischer), and donkey anti-rabbit Cy5 (1:200, Swant) secondary antibodies. After immunohistochemical processing, sections were adhered to gelatinized slides and mounted with Fluoromount G (Southern Biotech). To assess antibody specificity, control samples were processed in parallel as described but without primary antisera.

### Intraepidermal nerve fiber quantification

Footpad samples were viewed under an Olympus BX-51 microscope equipped for epifluorescence using appropriate filters. For the quantitative analyses of IENF, individual fibers were counted as they pass through crossed the basement membrane. Branching occurring within the epidermis was not considered. IENF were counted at the lateral flat side of the footpad and expressed as number of fibers per 1 mm length of epidermis using the epifluorescence microscope. The average density of IENF per animal was then derived for IENF counting. IENFs were also counted for CGRP and IB4 intraepidermal positive fibers separately. 4 mice per age and group were analyzed. Confocal images were also taken with a Leica microscope.

### Sweat gland nerve fiber density

Digital images of the sweat glands of the footpad of each animal were obtained with the epifluorescence microscope. Nerve fibers in the gland were selected using computer assisted image analysis (ImageJ software), and the SGNF density was estimated as the percent area of nerve fibers within the area of interest^25,26^. The area occupied by single glands (area of interest) was manually delineated, and the VIP-labeled nerve fibers within were quantified subtracting background based on the pixels above threshold, using ImageJ software. Using VIP channel only, background thresholding was performed using automatic threshold plugin method, based on a grayscale image. A mean of 19 glands per animal were analyzed, and for each animal (WT and SOD1^G93A^, at 4 and 12 weeks of age, 4 mice per age and group) we calculated the median percent area of SGNF.

### DRG sample processing and immunohistochemical procedure

L4 and L5 DRG were extracted from wild type and SOD1^G93A^ mice at 4, 8, 12 and 16 weeks (4 mice per age and group). DRG were maintained at 4 °C in a cryoprotective solution and then were embedded in OCT (optimal cutting temperature compound) and serially cut with a cryotome in 20-µm thick sections, collecting the slices in gelatinized slides.

For immunofluorescence, slides were blocked as above and incubated in lectin (unconjugated Griffonia Simplicifolia Lectin I) at 4 °C overnight, and subsequently incubated overnight at 4 °C with anti-PGP9.5, anti-CGRP, anti-parvalbumin (1:1000, PV-28 Swant) and anti-lectin. After washes, sections were incubated overnight at 4 °C with Alexa Fluor 488, donkey anti-rabbit Cy5, donkey anti-mouse Alexa Fluor 594 as secondary antibodies. DAPI staining was also used to ensure correct recognition of tissue structures. Slides were mounted in Fluoromount G (Sourthern Biotech). To assess antibody specificity, control samples were processed in parallel as described but without primary antibodies.

### DRG sensory neurons quantification

Photographs of the entire area of the DRG were taken using a confocal microscope. Every second serial section from the DRG was analyzed (with a distance of 20 μm between sections), and a total of 6 sections was analyzed for each DRG (comprising a total thickness of 320 μm, as representative area of the ganglia). Each image of the entire DRG was a composite of individual images. The number of immune-positive DRG neurons was determined counting neurons that contained a nucleus and showed a strong signal intensity in the cytoplasm. Area of these DRG sections was also calculated and the density of neurons per mm^2^ was derived. The total number of neurons (labeled for PGP9.5), as well as the number of CGRP+, PV+, IB4+ and double IB4+/CGRP+ neurons were counted. Percentage of each neuronal class was then derived.

### Data analysis

Data are expressed as mean ± SEM. Means were compared by two-way ANOVA applying Tukey’s post hoc test when necessary (SPSS statistics 25 software). The level of significance was set at *p* < 0.05.

## Results

### Intraepidermal nerve fiber density

**Figure 1:**
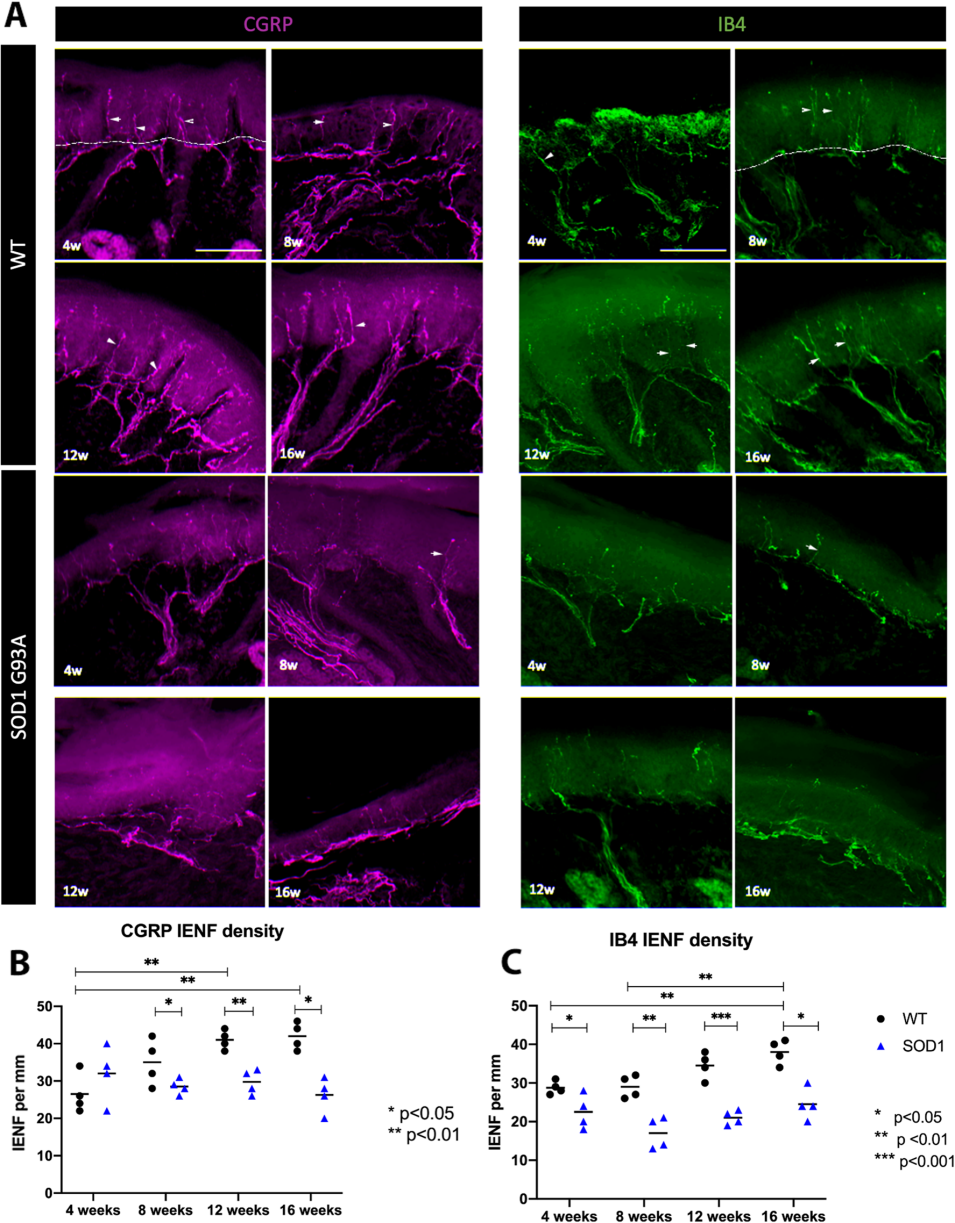
IENF density through time of both sensory epidermal populations in SOD1^G93A^ mice. (**A**) representative microphotographs of epidermal innervation of CGRP+ and IB4+ fibers of WT and SOD1 mice, at 4, 8, 12 and 16 weeks*.* Immunofluorescence colours have been adapted for the colour-blind. Scale bar: 100 μm. Arrowheads point to IENF; a dashed line overlies the basement membrane separating dermis and epidermis. (**B**) Plots representing peptidergic (CGRP+) and (**C**) non-peptidergic (IB4+) IENF density. 4 mice WT (black dots) and 4 mice SOD1^G93A^ (blue triangles) were used in each group. Differences were assessed by two-way ANOVA with post hoc analysis. Both peptidergic and non-peptidergic intraepidermal axons density increase over time in the WT, while in the SOD1^G93A^ mice remain stable. CGRP+ fibers were significantly lower in SOD1^G93A^ compared to WT mice at 8, 12 and 16 weeks of age, whereas IB4+ fibers were reduced already at 4 weeks of age. *IENF* intraepidermal nerve fiber.

We quantified IENF density of two populations of sensory neurons: nociceptive peptidergic (CGRP+) and non-peptidergic (IB4+) (Fig. 1A). In the WT mice, both peptidergic and non-peptidergic fibers density showed an increase with age. In the WT mice, the mean number of CGRP+ and IB4+ axons at 4 weeks of age were 27 ± 5 and 29 ± 1 per mm of epidermal length respectively and increased progressively to 42 ± 3 and 38 ± 4 respectively at 16 weeks (Fig. 1B,C). This increase was statistically significant from 4 to 16 weeks (p = 0.002) and from 4 to 12 weeks (p = 0.004) in the peptidergic fibers, and from 4 to 16 weeks (p = 0.003) and from 8 to 16 weeks (p = 0.004) in the IB4+ axons. The density of peptidergic fibers was slightly higher than that of the non-peptidergic fibers.

In the SOD1 transgenic mice both populations of IENF remained stable overtime, and again the peptidergic fibers were marginally higher than the non-peptidergic ones. We found a lower density of intraepidermal IB4+ fibers (22 ± 2 fibers per mm, p = 0.034) in the 4 weeks SOD1^G93A^ mice as compared with corresponding WT mice, but not in the CGRP+ population (31.6 ± 2.7 fibers per mm, p = 0.396). At 8, 12 and 16 weeks of age, the SOD1^G93A^ mice presented significantly lower density of both subpopulations of IENF compared to the WT mice (p < 0.05) (Fig. 1B,C).

### Sweat gland innervation

**Figure 2:**
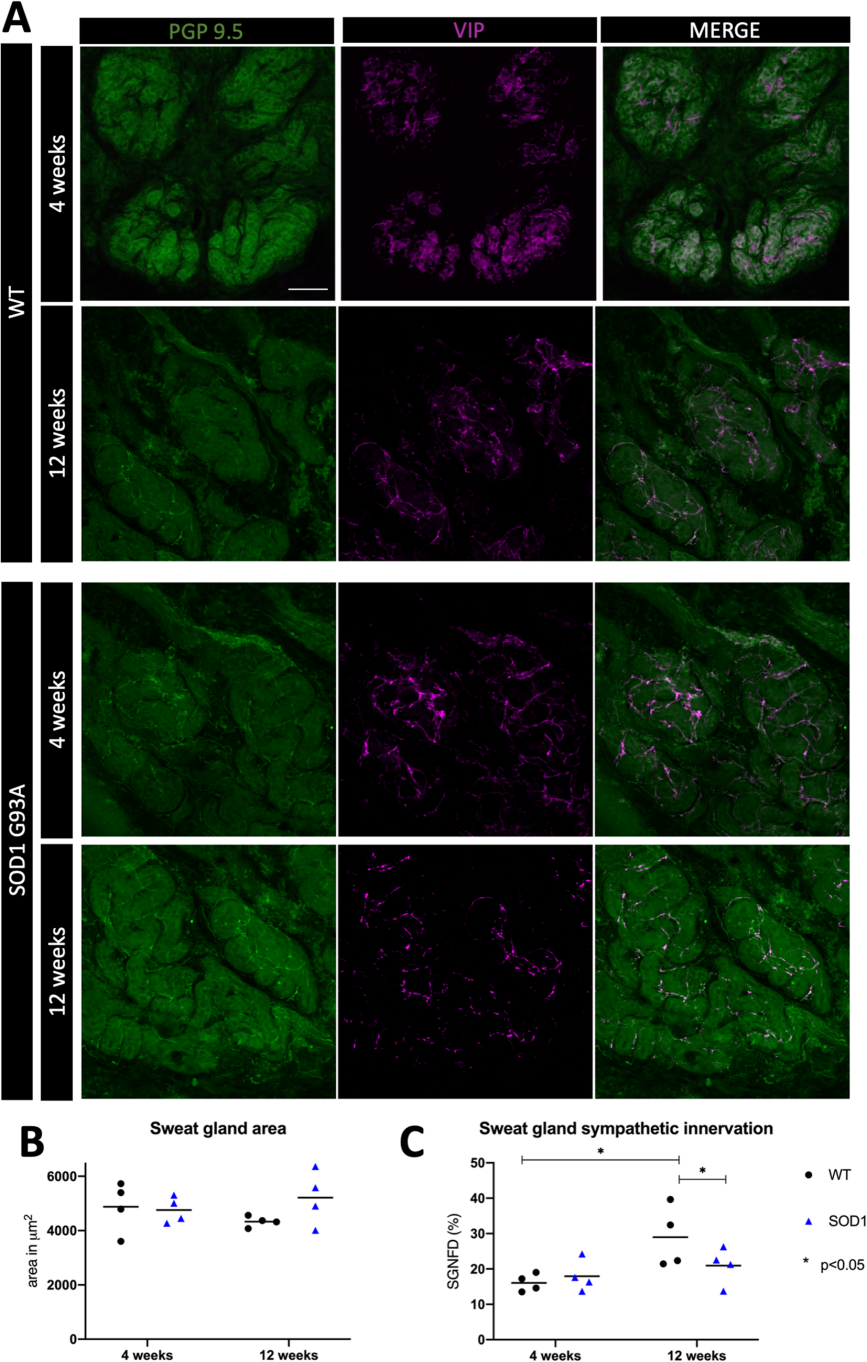
Sweat gland nerve fiber innervation is decreased in the SOD1^G93A^ mice. (**A**) Representative microphotographs of sweat gland innervation, labele for PGP9.5 and for VIP in SOD1^G93A^ and WT mice at 4 and 12 weeks. Immunofluorescence colours have been adapted to the colour-blind. (**B**) The mean sweat gland area remained stable overtime in both SOD1^G93A^ and WT mice. (**C**) SGNF density was similar in SOD1^G93A^ and WT mice at 4 weeks of age, but at 12 weeks, it was significantly lower in the SOD1^G93A^ mice. Scale bar: 50 μm. 4 mice WT (black dots) and 4 mice SOD1^G93A^ (blue triangles) were used in each group. Differences were assessed by two-way ANOVA with post hoc analysis. *p < 0.05. *SGNFD* sweat gland nerve fiber density.

At 4 weeks (presymptomatic stage), sweat gland innervation was similar between the SOD1^G93A^ and the WT mice. However, at 12 weeks, while there was an increase of SGNF density in the WT mice (p = 0.029), in the SOD1^G93A^ mice did not change. At 12 weeks, SGNF density in the SOD1^G93A^ mice was 29% lower than in the WT (p < 0.05). We also analyzed sweat gland size and did not find differences between the two groups of mice neither in the presymptomatic nor in the symptomatic stage (Fig. 2).

### Dorsal root ganglion sensory populations

**Figure 3:**
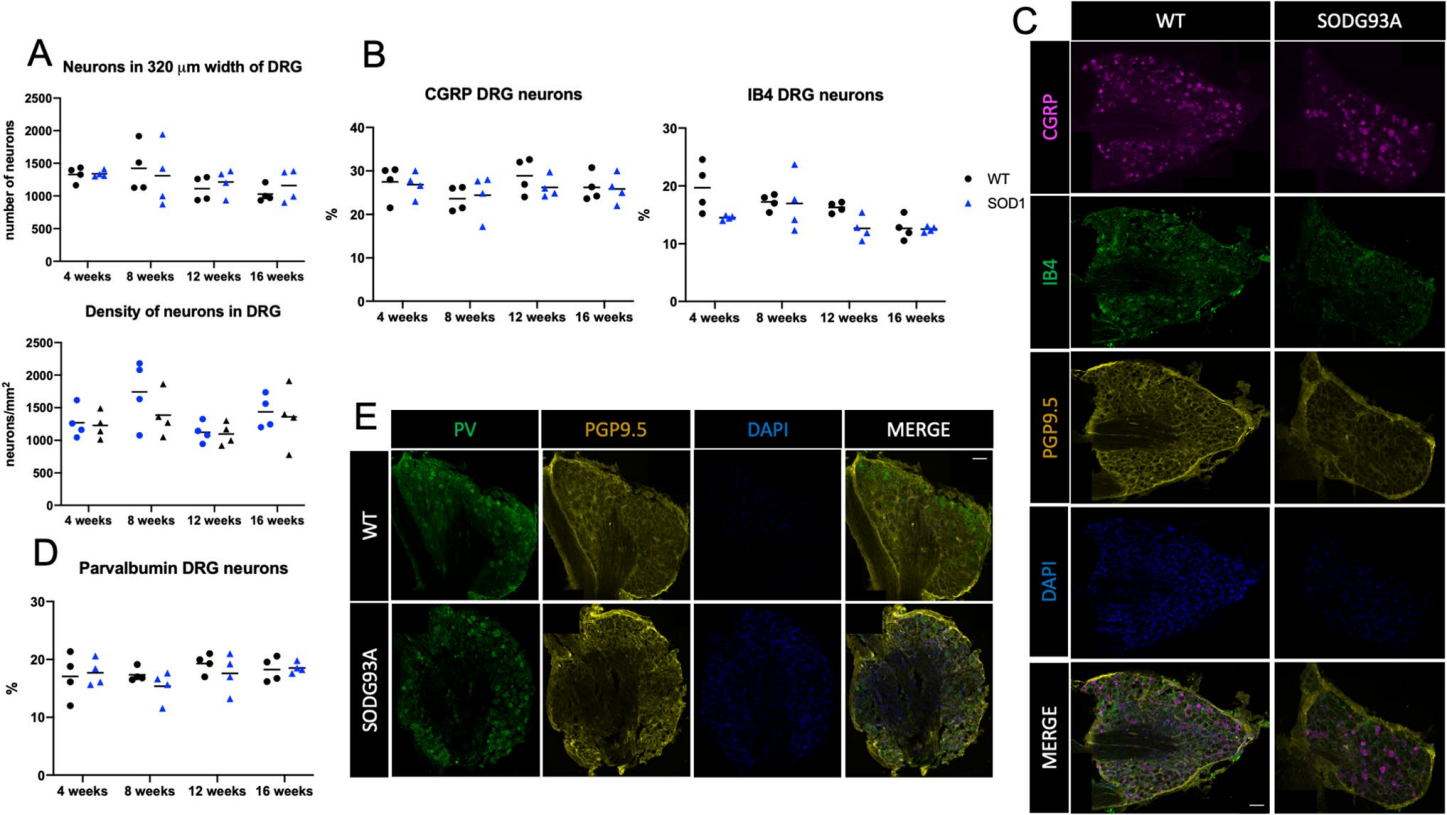
Neuronal bodies are preserved in the DRG of the SOD1^G93A^ mice. (**A**) Total number of neurons in 320 μm width of the DRG (counted in 6 sections of the DRG) (top plot), and density of neurons per mm^2^ in the DRG (bottom plot). There were no differences in the overall number nor in the density of DRG neurons. PGP9.5 labeling was used for the overall neuron count. (**B**) Percentage of CGRP and IB4 sensory populations in the DRG of SOD1^G93a^ mice compared to the WT. There were no differences between groups and overtime. (**C**) Representative images of DRG of WT and SOD1^G93A^ mice showing CGRP and IB4 sensory populations. Scale bar: 100 μm. (**D**) Percentage of PV+ DRG sensory populations of SOD1^G93A^ mice compared to the WT. There were no differences between groups and overtime. (**E**) Representative images of DRG of WT and SOD1^G93A^ mice showing PV sensory neurons. Scale bar: 100 μm. Immunofluorescence colors have been adapted to the color-blind. 4 mice WT (black dots) and 4 mice SOD1^G93A^ (blue triangles) were used in each group. Differences were assessed by two-way ANOVA with post hoc analysis.

Firstly, to ensure that the percentage comparisons of the different sensory populations were not affected by neuronal loss, we quantified the total number of neurons in 6 sections covering a 320 μm thickness of each DRG, a representative volume of the DRG, and the density of total neurons per mm^2^ in the sections studied, of each mouse at each stage. We did not detect significant differences between groups at any age, neither in the total number of neurons nor in the density of neurons (Fig. 3A). Then, we calculated the percentage of IB4+, CGRP+ and PV+ positive neurons of the total DRG neurons of SOD1^G93A^ mice and WT littermates (Fig. 3C,E).

In the WT mice, CGRP+ neurons represented 26.9% (IQR 24.2–30.1), IB4+ 16.8% (IQR 15.2–17.9), and PV+ 17.8% (IQR 16.2–19.6) of total DRG neurons. A small fraction of neurons (1.26%, IQR 0.89–2.2) presented double labelling for CGRP and IB4. In the SOD1^G93A^ mice the results were similar: CGRP+ 26.4% (IQR 24.8–27.7), IB4+ 14.3% (IQR 12.2–15.2), PV 18.0% (IQR 15.6–18.5), and CGRP+/IB4+ 0.86% (0.6–1.05). Therefore, the number of DRG neurons and the estimated proportions of CGRP+, IB4+ and PV+ did not change overtime and did not show differences between the WT and the SOD1^G93A^ mice (Fig. 3B,D).

## Discussion

In this study we found an early impairment of IENF of both peptidergic and non-peptidergic neurons, and a later impairment of sudomotor fibers in the SOD1^G93A^ mice compared with their WT littermates. In the WT mice, the density of peptidergic and non-peptidergic IENF showed an increase from 4 to 16 weeks of age, as well as the VIPergic sweat gland innervation^25^. In contrast, in the SOD1^G93A^ mice the corresponding densities remained steady or with slight, non-significant decline. Early pathological evidence has been already identified in the muscle innervation in SOD1^G93A^ mice. Thus, at 4 weeks, virtually all motor end-plates in hindlimb muscles were innervated, but significant denervation of end-plates was detected already by 7 weeks and continued to progress until time of death^27^. This was paralleled by a significant decline in the amplitude of motor compound action potentials in the same muscles found from 8 weeks of age^24^.

Although at advanced stages of the disease the involvement of both populations of sensory fibers labeled was similar, initially IB4+ fibers were the most affected. Different factors may contribute to the different vulnerability between peptidergic and non-peptidergic axons in the early stage of the disease. It is worth mentioning that such differences are also seen after peripheral nerve damage, when IB4+ projections in the dorsal horn tend to regress, while CGRP+ projections are less affected^28–30^. IB4 neurons lack α7-integrin, a laminin receptor essential for optimal axonal regeneration in the peripheral nervous system, while it is expressed in CGRP and NFH (neurofilament heavy-chain) neurons^31^. Also, cultured DRG IB4 neurons, although they respond to GDNF, exhibit a lower neurite outgrowth compared to other sensory neuronal populations^32^. In line with this, although after a peripheral nerve lesion NGF and GDNF are both upregulated^33–36^, the latter shows lower levels in the DRG^32,37^. In the SOD1^G93A^ mice, GDNF mRNA expression in the hindlimb muscles is indetectable at the presymptomatic stage, increases by the onset of motor symptoms and continues with a marked decrease overtime, while in ALS patients GDNF mRNA expression tends to reduce in advanced muscle pathology, and the serum levels of GDNF are markedly lower in ALS patients than in healthy subjects^38–40^. In the skin of adult animals, GDNF and NGF are also both expressed, but in the case of GDNF at low levels, and changes of its expression after tissue damage or inflammation are unclear^40^. In contrast, NGF is clearly increased after skin injury^41,42^, causing collateral axonal sprouting and contributing to nociceptor sensitization and hyperalgesia^43,44^. Therefore, it seems that although at advanced stages both sensory fiber populations are similarly affected, intrinsic difficulties of IB4 neurons for axonal growth and sprouting may explain their earlier susceptibility. Additional factors may be considered to explain the reduced density of epidermal axons since, for example, SOD1^G93A^ have weight loss in symptomatic stages compared to the WT^45^.

In addition to the IENF involvement, our findings also show a reduction of sweat gland innervation in the SOD1^G93A^ mice. This observation is in correspondence with reports in ALS human patients showing involvement of unmyelinated postganglionic sympathetic fibers and sweating deficiencies^46–48^. Axons innervating the sweat glands show degeneration, TDP-43 accumulation, increased intracytoplasmic granules and synaptic vesicles accumulation, in parallel to decrease of sympathetic pilomotor fibers density^6,46,49^.

Whereas the skin innervation showed a reduced number of IENF, affecting both peptidergic and non-peptidergic fibers (present results) and also reduced innervation of Meissner corpuscles in SOD1^G93A^ mice^9^, DRG analyses showed that different sensory populations are preserved, with only a marginal decrease of double positive neurons (CGRP+ and IB4+) at 12 weeks. These findings are in line with previous work that studied two sensory neuron populations in the DRG of SOD1^G93A^ mice^50^, and also reported no differences of nociceptive and mechanoreceptor/proprioceptor sensory neuronal populations between the mutant mice and the WT at 30 and 100 days of age.

Additionally, regarding proprioceptors, degeneration of sensory Ia/II fibers of the muscle spindles has been observed in SOD1^G93A^ and TDP43^A315T^ mutated mice already at presymptomatic stage^51^, despite there is no loss of proprioceptive neurons in the DRG. Therefore, the loss of distal axons with preservation of the neuronal soma suggests that distal axonopathy is the main physiopathological mechanism involved in the sensory involvement. A motor distal axonopathy has already been proposed based on the findings of early loss of neuromuscular junctions in both animal and human ALS, before the onset of clinical symptoms, and even before motor neuron loss in the anterior horn of the spinal cord^52–56^.

Despite there is no evidence of significant neuronal loss in the DRG of ALS mice, it has been reported the occurrence of pathological changes such as cytoplasmic fragmentation, microvacuolization, macrophage and microglia recruitment^57^ and swollen mitochondria^58^. Also, SOD1 protein accumulation has been observed in PV + neurons, but not in CGRP, IB4 and SP neurons of the SOD1^G93A^ mouse. However, it has been described a loss of 53% of dorsal root axons^59^ and signs of Wallerian degeneration^58^, that could be due to particular vulnerability of the sensory axons. Other studies have confirmed sympathetic preganglionic neuronal loss in the intermediate lateral column (IML) of the spinal cord^60,61^ but characteristically with preservation of sympathetic ganglion cells^62^.

There is an increased amount of evidence regarding the role of axon homeostasis in the physiopathology of ALS: from mitochondrial dysfunction, increase in the oxidative stress radicals and deficits in axonal transport. Several genes involved in motor neuron disease in general, and in ALS in particular, are tightly involved in axonal transport^63^. Indeed, in ALS mouse models, deficit in both anterograde and retrograde transport are reported as early events^64,65^. Moreover, TDP-43 protein, a hallmark of both sporadic and familial forms of ALS, may affect the axonal transport^66^, and can contribute to protein aggregation through axonal translational disturbances^67^. Despite we cannot completely discard that the deficit of the cutaneous innervation in the SOD1^G93A^ mouse could be in part due to distal degeneration of the thin sensory and sudomotor fibers, it seems most likely compatible with a reduced capacity of the terminal axons to grow and mature during young age, since the values found at 4 weeks did not increase as in WT mice. Indeed, the capability of axonal regeneration in the SOD1^G93A^ mouse has been reported to be affected already in young animals, showing reduced number of regenerated fibers, slower recovery and increased motoneuron death after sciatic nerve injury^68,69^. It is worth to note that regenerating axons reaching the skin have difficulties to cross the basement membrane and few grow more than half-way in the epidermis^70,71^.

## Conclusions

In summary, we have found that sensory involvement in the SOD1^G93A^ mouse is located in the distal part of the axon, affecting all types of small nerve fibers, and also sympathetic sudomotor fibers. There are slight differences in the rate of IENF reduction that could translate an intrinsic susceptibility for maintenance and growth. Furthermore, analysis of peripheral axonal endings and their neuronal bodies in the DRG, confirm that while there is a significant impairment of the distal part of the axons, their somas are preserved, suggesting a distal sensory axonopathic mechanism, similarly to what was confirmed in the motor counterpart of the disease.
